# A novel approach to control *Botrytis cinerea* fungal infections: uptake and biological activity of antifungals encapsulated in nanoparticle based vectors

**DOI:** 10.1038/s41598-022-11533-w

**Published:** 2022-05-14

**Authors:** Giulia De Angelis, Giovanna Simonetti, Laura Chronopoulou, Anastasia Orekhova, Camilla Badiali, Valerio Petruccelli, Francesca Portoghesi, Simone D’Angeli, Elisa Brasili, Gabriella Pasqua, Cleofe Palocci

**Affiliations:** 1 Department of Environmental Biology, “Sapienza” University of Rome, P. le Aldo Moro 5, 00185 Rome, Italy; 2 Department of Chemistry, “Sapienza” University of Rome, 00185 Rome, Italy; 3 Department of Public Health and Infectious Diseases, “Sapienza” University of Rome, 00185 Rome, Italy

**Keywords:** Nanoparticles, Antifungal agents

## Abstract

*Botrytis cinerea*, responsible for grey mold diseases, is a pathogen with a broad host range, affecting many important agricultural crops, in pre and post harvesting of fruits and vegetables. Commercial fungicides used to control this pathogen are often subjected to photolysis, volatilization, degradation, leaching, and runoff during application. In this context, the use of a delivery system, based on poly (lactic-co-glycolic acid) nanoparticles (PLGA NPs) represents an innovative approach to develop new pesticide formulations to successfully fight *B. cinerea* infections. In order to study NPs uptake, *B. cinerea* conidia and mycelium were treated with PLGA NPs loaded with the high fluorescent probe coumarin 6 (Cu6-PLGA NPs) and analyzed under ApoTome fluorescence microscopy. The observations revealed that 50 nm Cu6-PLGA NPs penetrated into *B. cinerea* conidia and hyphae, as early as 10 min after administration. Pterostilbene, a natural compound, and fluopyram, a synthetic antifungal, were entrapped in PLGA NPs, added to *B. cinerea* conidia and mycelium, and their antifungal activity was tested. The results revealed that the compounds loaded in NPs exhibited a higher activity against *B. cinerea.* These results lay the foundations for the use of PLGA NPs as a new strategy in plant pest management.

## Introduction

Fungal plant pathogens are the main cause of considerable economic losses of crop plants^1^. Ascomycete *Botrytis cinerea*, responsible for grey mold diseases, is a highly successful pathogen due to its flexible infection modes, high reproductive output, wide host range, and ability to survive for extended periods as conidia and/or small hardened mycelia masses called sclerotia^2^. It is a pathogen with a broad host range, affecting many important agricultural crops, mainly in pre and post harvesting of fruit or vegetables. It is estimated that *B. cinerea* causes a $10 to 100 billion of production loss annually worldwide^3^. Due to the highly destructive nature of *B. cinerea*, it was ranked second on a list of fungal pathogens of scientific and economic importance^4^. Approximately 8% of the global fungicide market is used to control this pathogen. It is important to highlight that less than 0.1% of fungicides reach their biological targets as 90% of them are lost through photolysis, volatilization, degradation, leaching, and runoff during application^5^. In addition, fungicide usage is harmful to both the environment and human health, and *B. cinerea* has developed resistance to many conventional fungicides such as dicarboximides and benzimidazoles^6^.

To overcome the fungal multi-resistance to existent drugs, it is important to explore novel antifungal agents, which may replace current control strategies. Some authors reported the activity of plant extracts or natural compounds against *B. cinerea*^7^. In this context, the generation of drug delivery systems based on nanomaterials represents a potential alternative to develop novel formulations to successfully combat fungal infections and overcome the fungal multi-resistance to existent drugs^8^.

Different studies have determined that NPs present fewer side effects and greater specificity to the infection site^9,10^. Special attention has been given to biopolymeric nanoparticles (NPs) as non-toxic and eco-friendly nanocarriers that can be successfully used for the controlled release of bioactive compounds^11^. In recent years, a variety of natural and synthetic polymers have been explored for nanoformulations. Among them, polylactic acid (PLA), polyglycolic acid (PGA), and their copolymer (PLGA) have been extensively investigated due to their biocompatibility, high solubility, stability, and effectiveness^12–15^.

It has been shown that PLGA NPs have the ability to penetrate *Vitis vinifera* cell suspensions without harmful effects on cellular vitality^16,17^. As largely investigated by the authors in *V. vinifera* cell cultures, methyl jasmonate (MeJA) encapsulation in PLGA NPs significantly promoted MeJA cell uptake and the activation of MeJA-induced responses^13^. Simonetti et al.^15^ have demonstrated the anti-*Candida* biofilm activity of PLGA NPs loaded with non-fermented grape pomace.

Up to now, there is relatively little and limited evidence about PLGA NPs uptake by pathogenic fungal cells^18,19^, and further studies are needed in order to improve the knowledge on the effectiveness of PLGA NPs to deliver natural or conventional antifungals to microbial cells.

The present research aimed to study PLGA NPs uptake in *B. cinerea* and investigate the antifungal activity of natural and synthetic compounds entrapped in PLGA NPs against *B. cinerea* conidia and mycelium.

In order to examine NPs uptake*, B. cinerea* conidia and mycelium were treated with PLGA NPs loaded with the high fluorescent probe coumarin 6 (Cu6-PLGA NPs) and analyzed under ApoTome fluorescence microscopy. Moreover, in an effort to investigate the antimicrobial activity of antifungals delivered by NPs, pterostilbene and fluopyram were encapsulated in PLGA NPs and administered at different stages of *B. cinerea* development.

Pterostilbene is a natural antimicrobial phenolic compound derived from resveratrol, mostly contained in *V. vinifera* leaves and grape berries. It has been demonstrated that pterostilbene has no harmful effects on plant metabolism or crop yield, and it is able to inhibit the growth of several phytopathogenic fungi, such as *Leptosphaeria maculans*, *Peronophythora litchii*, *Botrytis cinerea* and others^20,21^. Schmidlin et al.^22^ showed that pterostilbene was 5 to 10 times more effective than resveratrol in inhibiting the germination of conidia of *B. cinerea* and sporangia of *P. viticola*. On the other hand, fluopyram is a synthetic fungicide and nematicide compound commonly used in agriculture against *B. cinerea*.

This study provides new evidence of the use of PLGA NPs as an interesting strategy in integrated plant disease management, with the aim to increase the potency and efficiency of natural and conventional antifungals through a controlled and targeted drug release while decreasing environmental toxicity and agricultural costs.

## Materials and methods

### Chemicals and materials

Pterostilbene was purchased from Chemodex (St. Gallen, Switzerland). Poly(d,l)-lactic-co-glycolic acid (PLGA, lactide: glycolide 50:50, MW 50 kDa), coumarin 6 (Cu6) (98%), potato dextrose agar (PDA), fluopyram, RPMI medium (RPMI 1640 with l-glutamine, without bicarbonate), MOPS acid, XTT [2,3-bis-(2-methoxy-4-nitro-5-sulfophenyl)-5-(carbonyl (phenylamino)]-2H-tetrazolium hydroxide] and menadione (MEN) were purchased from Sigma-Aldrich (Milan, Italy).

Potato dextrose broth (PDB) was purchased from Formedium LTD (Hunstanton, Norfolk, England).

The microfluidic flow focusing reactor was assembled by the research group involved in the study as reported previously by Bramosanti et al.^23^.

### Synthesis of PLGA NPs

PLGA NPs loaded with fluopyram, pterostilbene or coumarin 6 were prepared by using an innovative microfluidic reactor with a flow-focusing configuration described previously^24^. The reactor consists of three inlets and one main mixing outlet channel. An organic phase containing the polymer is injected into the middle channel and water is injected into the two side inlets. NPs formation occurs through a nanoprecipitation mechanism in the mixing channel and NPs can be recovered at its end. PLGA (2 mg mL^−1^) and the selected payload were dissolved in an organic phase. Acetone was used to dissolve pterostilbene (1 mg mL^−1^) and coumarin 6 (40 μg mL^−1^), while fluopyram (0.2 mg mL^−1^) was dissolved in DMSO. To optimize the amount of encapsulated fluopyram and pterostilbene, PLGA NPs were prepared with different polymer/drug ratios, respectively from 2.5:1 to 20:1 and from 2:1 to 4:1, keeping PLGA concentration constant. A PLGA/coumarin 6 ratio of 50:1 was chosen on the basis of previous works^24^. The aqueous flow rate was 2000 µL min^−1^ while the organic phase flow rate was 100 μL min^−1^ when using acetone and 400 µL min^−1^ when using DMSO. The formed PLGA-based NPs were recovered and the organic phase was eliminated under reduced pressure. The NPs aqueous suspensions were stored at 4 °C until use.

### PLGA NPs characterization

Dynamic light scattering (DLS) measurements were carried out using a NanoZetasizer (Malvern Instruments, Malvern, UK) to measure the mean hydrodynamic diameter of PLGA NPs and their polydispersity index. The experimental conditions used are the following: a helium neon laser operating at 633 nm, a fixed scattering angle of 173° and constant temperature (25 °C). The measured autocorrelation functions of the scattered light intensity were analyzed using the CONTIN algorithm in order to obtain the decay time distributions^25^. Decay times were used to determine the distributions of the diffusion coefficients of the particles (D), converted in turn in the distributions of the apparent hydrodynamic radii, R_H_, using the Stokes–Einstein relationship: R_H_ = k_B_T/6πηD (kBT = thermal energy; η = solvent viscosity).

Particle morphology was observed by scanning electron microscopy (SEM) in both the secondary and the backscattered electron modes with an electron acceleration voltage of 20 keV, using a LEO 1450VP SEM microscope (ZEISS, Oberkochen, Germany).

The quantitative analysis of fluopyram and pterostilbene loaded in PLGA NPs was carried out by spectroscopic measurements. NPs aqueous suspensions were ultra-centrifuged at low temperature (4 °C) to recover NPs. The supernatant was discarded and the pellet was dissolved in DMSO and analyzed by measuring the UV absorbance at 270 nm (for fluopyram) or at 313 nm (for pterostilbene), comparing the results with the corresponding calibration curve. The method used for fluopyram was linear within the concentration range between 0.2 and 0.8 mg mL^−1^ with R^2^ = 0.997. The method used for pterostilbene was linear within the concentration range between 0.002 and 0.01 mg mL^−1^ with R^2^ = 0.9822.

The encapsulation efficiency and loading capacity were calculated by using the following equations:

$$\left( {EE\% } \right) = \frac{{\left( {Total\;drug\;added - free\;non\text{-}entrapped\;drug} \right)}}{{\left( {Total\;drug\;added} \right)}} \times 100$$

$$\left( {LC\% } \right) = \frac{{\left( {Amount\;of\;total\;entrapped\;drug} \right)}}{{\left( {Total\;nanoparticle\;weight} \right)}} \times 100$$

### In vitro release studies of fluopyram and pterostilbene from PLGA NPs

A fixed amount (2 mg) of fluopyram or pterostilbene-loaded PLGA NPs was suspended in 2 mL of acetate buffer at pH = 4 or in 2 mL of PBS solution at pH = 7.4 in a centrifuge tube. The suspensions were incubated at 25 °C and maintained under magnetic stirring at 300 rpm. At fixed time intervals, 500 µL of the supernatant was collected and replaced with an equal volume of buffer in order to keep the reaction volume constant. The concentration of fluopyram or pterostilbene in the collected supernatant was spectrophotometrically determined as reported above. At each time point, the amount of released drug was calculated by normalizing the data with the total amount of drug inside the particles.

### Fungal strain and culture condition

*Botrytis cinerea* DSM 877, obtained from the German Collection of Microorganisms (DSMZ, Braunschweig, Germany), was used in this study. This strain has been reported to show an intermediate pathogenicity^26^ and it was used for the evaluation of antifungal compounds^27^. The strain was cultured on potato dextrose agar (PDA) at 24 °C. Conidia were collected from 10-day-old and the concentration was determined using a Thoma counting chamber. RPMI medium (RPMI 1640 with l-glutamine, without bicarbonate) buffered to pH 7.0 with 0.165 M MOPS was used for antifungal tests. XTT [2,3-bis-(2-methoxy-4-nitro-5-sulfophenyl)-5-(carbonyl (phenylamino)]-2H-tetrazolium hydroxide] and Menadione (MEN) were used to test the metabolic activities of *B. cinerea* cells.

### Fungal uptake of Cu6-PLGA NPs

The aqueous suspension of Cu6-PLGA NPs was added to *B. cinerea* conidia and mycelium. In particular, *B. cinerea* conidia (1 × 10^5^ conidia mL^−1^) were inoculated into potato dextrose broth (PDB) and incubated at 24 °C. After 12 h Cu6-PLGA NPs, at a final concentration of 0.1 mg mL^−1^, were added. After 0, 10, and 60 min, the fungal suspension was placed on a microscope slide, and observed under ApoTome fluorescence microscope. For Cu6-PLGA NPs uptake in *B. cinerea* mycelium, *B. cinerea* (1 × 10^5^conidia mL^−1^) was cultured on glass microscope slides placed into Petri dishes containing PDB. After 24 h of incubation the medium was removed and the Cu6-PLGA NPs, at a final concentration of 0.1 mg mL^−1^, were added. After 0, 10, and 60 min, each glass microscope slide was removed from the Petri dish and observed under ApoTome fluorescence microscope. A control of untreated conidia and mycelium was observed to reveal any auto-fluorescence.

### Fluorescent analysis

Cu6-PLGA NPs treated conidia and mycelium were observed and images were acquired using an Axio Imager M2 fluorescence microscope (Zeiss, Germany), motorized on the 3 axes, using a FITC filter (λ excitation BP 455–495 nm; λ emission BP 05–555 nm). The high thickness of the sample required a Z-stack image scanning performed with an Axiocam 512 (Zeiss) monochromatic camera and ApoTome 2 (Zeiss) as fringe projection module used to remove the out of focus signal. Single plane images were obtained as Z-stack maximum projection using Zen 2.5 (Zeiss) image analysis software.

### In vitro antifungal activity of pterostilbene and fluopyram

In vitro antifungal activity was carried out as previously described by Meletiadis et al.^28^. *B. cinerea* conidia were grown in 96-well plates with fluopyram or pterostilbene, free or entrapped in PLGA NPs. After 0, 1, 5, 24, 48, and 72 h of incubation at 24 °C, XTT-menadione were added to each well to obtain final concentrations of 200 μg mL^−1^ for XTT and 25 μM for menadione. The microtitration plates were incubated for a further 2 h at 24 °C and the optical density at 450 nm (OD450) was measured. A minimum of four replicates was performed. The percentage of MA inhibition (100% − %MA) was compared with the drug-free control.

### Statistical analysis

The data were expressed as mean ± SD, and P < 0.05 was considered statistically significant.

Statistical criteria, p, and other parameters are shown for each experiment. The statistical data analysis was performed using the GraphPad Prism 8 software (GraphPad Software Inc., USA).

## Results and discussion

### NPs characterization

**Figure 1:**
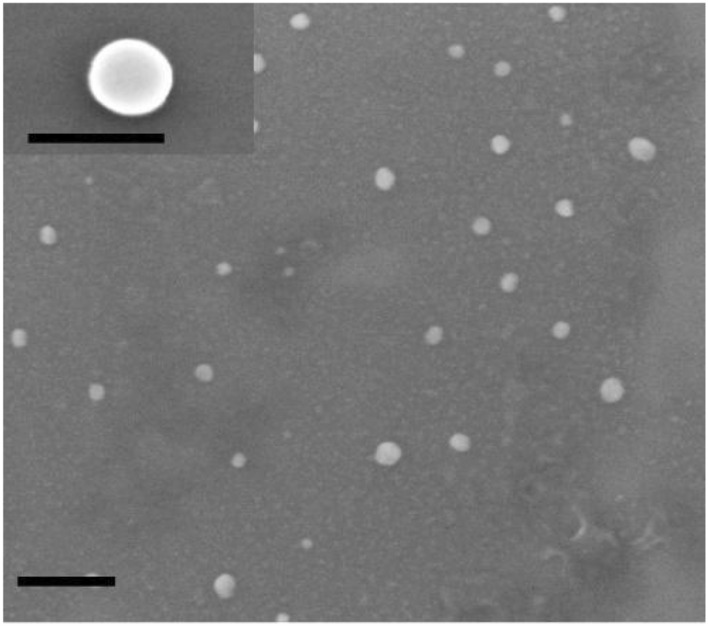
SEM micrograph of PLGA based NPs, dimension bar 200 nm (100 nm in the enlarged image).

PLGA NPs dimensions, characterized by DLS measurements, are reported in Table 1. According to the used experimental conditions, PLGA NPs entrapping fluopyram, pterostilbene, or coumarin 6, presented an average size of 50 nm, with polydispersity indexes below 0.2. Investigations by scanning electron microscopy (SEM) showed that drug-loaded PLGA NPs had a spherical morphology (Fig. 1). Drug loading efficiencies and loading capacities for fluopyram-PLGA NPs or pterostilbene-PLGA NPs prepared with different polymer/drug ratios are reported in Tables 2 and 3 respectively.

**Table 1:** Dimensions and Polydispersity Indexes (PdI) of PLGA-based NPs, measured by DLS.

NPs	Dimensions (nm)	PdI
Fluopyram-PLGA	47.95 ± 5.33	0.187 ± 0.06
Pterostilbene-PLGA	54.06 ± 7.64	0.119 ± 0.03
Coumarin 6 PLGA	48.48 ± 3.62	0.155 ± 0.02

Values are referred to as mean ± standard deviation.

**Table 2:** Drug loading efficiencies and loading capacities for fluopyram-PLGA NPs prepared with different polymer/drug ratios.

PLGA/fluopyram ratio (w/w)	Encapsulation efficiency (%)	Loading capacity (%)
20:1	64 ± 1	3.2 ± 0.05
15:1	83 ± 3	5.5 ± 0.03
10:1	89 ± 1	8.9 ± 0.01
8:1	78 ± 2	9.8 ± 0.05
5:1	56.3 ± 1	11.3 ± 0.1
2.5:1	37.5 ± 2	15.0 ± 0.6

**Table 3:** Drug loading efficiencies and loading capacities for PTB-PLGA NPs prepared with different polymer/drug ratios.

PLGA/pterostilbene ratio (w/w)	Encapsulation efficiency (%)	Loading capacity (%)
4:1	37 ± 2	9.2 ± 0.5
2:1	75 ± 3	37.5 ± 1.5

The PLGA/fluopyram and PLGA/pterostilbene weight ratios that showed the best encapsulation efficiency and loading capacity were respectively 10:1 and 2:1. These conditions were selected for all successive observations.

### In vitro release kinetics of fluopyram and pterostilbene from PLGA NPs

**Figure 2:**
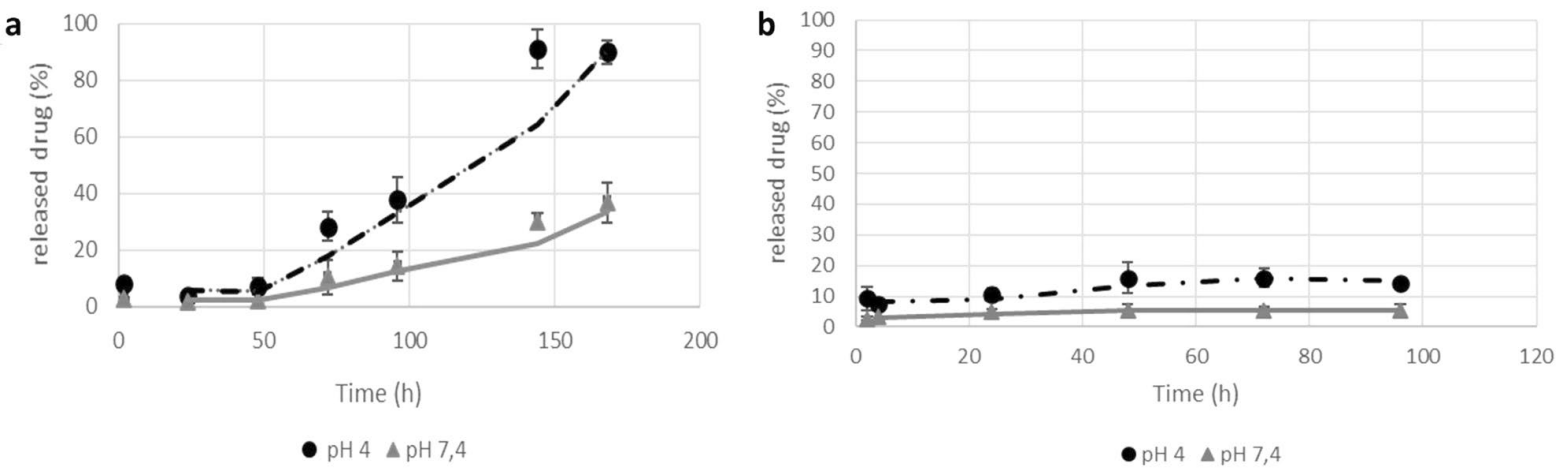
In vitro release kinetics of fluopyram (**a**) and pterostilbene (**b**) from PLGA NPs at 25 °C and different pH values.

The in vitro release of fluopyram occurs slowly reaching a plateau after 120 h as a function of medium pH (Fig. 2a). In both experimental conditions at pH 4 and 7.4, the quantity of released fluopyram results to be compatible with the in vitro inhibition protocols of *B. cinerea* (1.25–0.07 μg mL^−1^). Nevertheless, the release of the drug appears to be positively influenced by lower pH; in these experimental conditions, acid hydrolysis of the polymeric backbone could be favoured, which would influence the release of the drug by erosion. The amount of pterostilbene released within the time interval examined was low, probably due to the poor solubility of pterostilbene in water (0.011 g L^−1^) and the hydrophobic interactions that stabilize the PLGA-pterostilbene complex. No significant change in the amount of released pterostilbene as a function of pH was observed (Fig. 2b). The % of pterostilbene released at pH 4, at the plateau conditions, was equal to 16% and corresponded to 30 μg mL^−1^, an amount compatible with the in vitro inhibition protocols of *B. cinerea*, in which pterostilbene was used at different concentrations between 20 and 1.25 μg mL^−1^.

However, such results could be useful to compare in vitro and in vivo performances since in in vivo experiments other mechanisms that favor NPs erosion phenomena may occur.

### Microscopic observations of NPs uptake in *B. cinerea* conidia and mycelium

**Figure 3:**
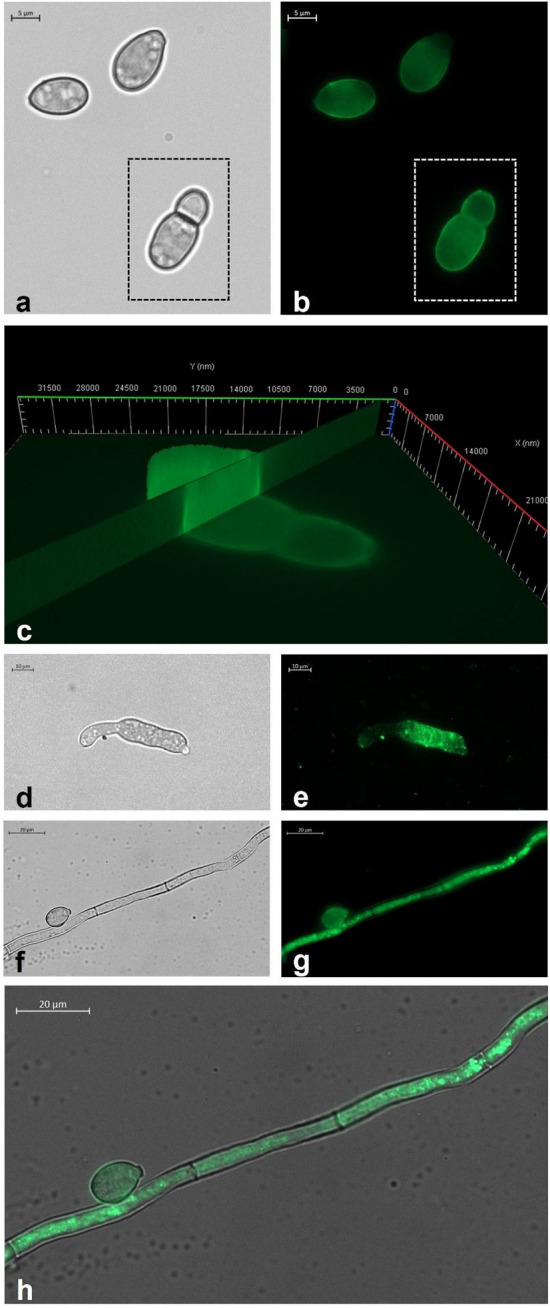
(**a**) Bright field and (**b**) fluorescence image of *B. cinerea* conidia treated for 10 min with Cu6-PLGA NPs. NPs fluorescence signal was evident in germinated *B. cinerea* conidia (white square) and non-germinated ones. (**c**) 3D reconstruction of *B. cinerea* conidia treated with NPs for 10 min. The fluorescence signal was detected inside conidia, along the whole thickness of the spore. (**d**) Bright field and (**e**) fluorescence image of *B. cinerea* germ tube. The NPs fluorescence signal was well visible in the expanding germ tube after 10 min of administration. (**f**) Bright field and (**g**) fluorescence image of *B. cinerea* conidium and hypha. The fluorescence signal was well visible both in the conidium and the hypha after 1 h of administration. (**h**) Overlap of the bright field image and the fluorescence image which shows the localization of Cu6-PLGA NPs inside the fungal conidium and hypha.

According to the microscopic analyses, Cu6-PLGA NPs administered to *B. cinerea* enter conidia and hyphae after 10 min. It was found that NPs interacted with the conidia wall from the first stages of germination before the formation of the septum (Fig. 3a,b). The conidia of *Botrytis cinerea* have relatively thick cell walls (0.5 μm) consisting of two layers, a thin, dark, rough outer layer that is electron-dense under the microscope, and a thicker inner one that is transparent under electron microscopy^29^. Based on fluorescence results, optical sections and the 3D reconstruction of the ApoTome Microscope, it has been possible to observe that the fluorescent signal was present both along the conidia wall and inside conidia, throughout the whole thickness of the spore (Fig. 3c). The NPs fluorescent signal was well visible also in the expanding germ tube (Fig. 3d,e). As the tube elongates, a large void forms in the center of the original spore.

The 50 nm Cu6-PLGA NPs were also administered to *B. cinerea* mycelium. 1 h after NPs administration, an intense signal appeared in the fungal hyphae (Fig. 3f–h). Fluorescence was visible in the mycelium after washing with sterile saltwater, showing that NPs penetrated or remained adhered to the hyphae. No autofluorescence was detected from the conidia and mycelium alone (data not shown). In a previous work, we demonstrated that PLGA NPs were able to penetrate the mycelium of some plant pathogenic fungi such as *A. carbonarius*, *A. niger* and *B. cinerea*^16^. During *B. cinerea* conidia germination, the outer spore wall breaks, and the emergent germ tube appears surrounded by the elastic inner one and by a mucilage sheath. At a very early stage, a transverse wall with a central pore is laid at the base of the germ tube^29^. Similarly, the uptake of Cu6-PLGA NPs has been previously demonstrated in fungal cells of *Aspergillus flavus.* In particular, Patel et al.^12^ showed that the uptake of Cu6-PLGA NPs in *A. flavus* spores and mycelium was closely related to NPs size. In such work, NPs of 203 nm interacted with fungal cell surfaces and were efficiently internalized after 1 h of incubation. Muse et al.^30^ observed that covalently tagged poly(lactic-co-glycolic) acid nanoparticles (PLGA-tetramethylrhodamine [PLGA-TRITC]), entrapping coumarin 6 (double-tagged) with a diameter of 85–150 nm released coumarin 6 in *A. flavus* spores and hyphae while the majority of the particles themselves did not seem to be trafficked into the interior of the cells. Nonetheless, some red fluorescence (PLGA-TRITC) was still observed within the interior of the cell, indicating that the smaller nanoparticles (30–50 nm) may have been directly endocytosed or diffused through the hyphae cell wall^30^. Other studies were carried out to test biopolymeric drug delivery systems against *B. cinerea*. In a recent work, Raj and collaborators^31^ showed that chitosan-arabic gum-coated liposome 5I-1H-indole nanoparticles were potent inhibitors against *B. cinerea* growth. Up to now, despite *B. cinerea* causing significant damage to agriculture every year, few data are available on its uptake of PLGA NPs. The inhibition of spore germination should be considered effective for controlling plant disease. In this work, we showed that PLGA NPs interacted and penetrated also in the conidia of *B. cinerea*, promoting the uptake of the encapsulated compound into fungal cells, with the aim to prevent spore germination and thus fungal infection.

### Antifungal activity of pterostilbene or fluopyram PLGA NPs

**Figure 4:**
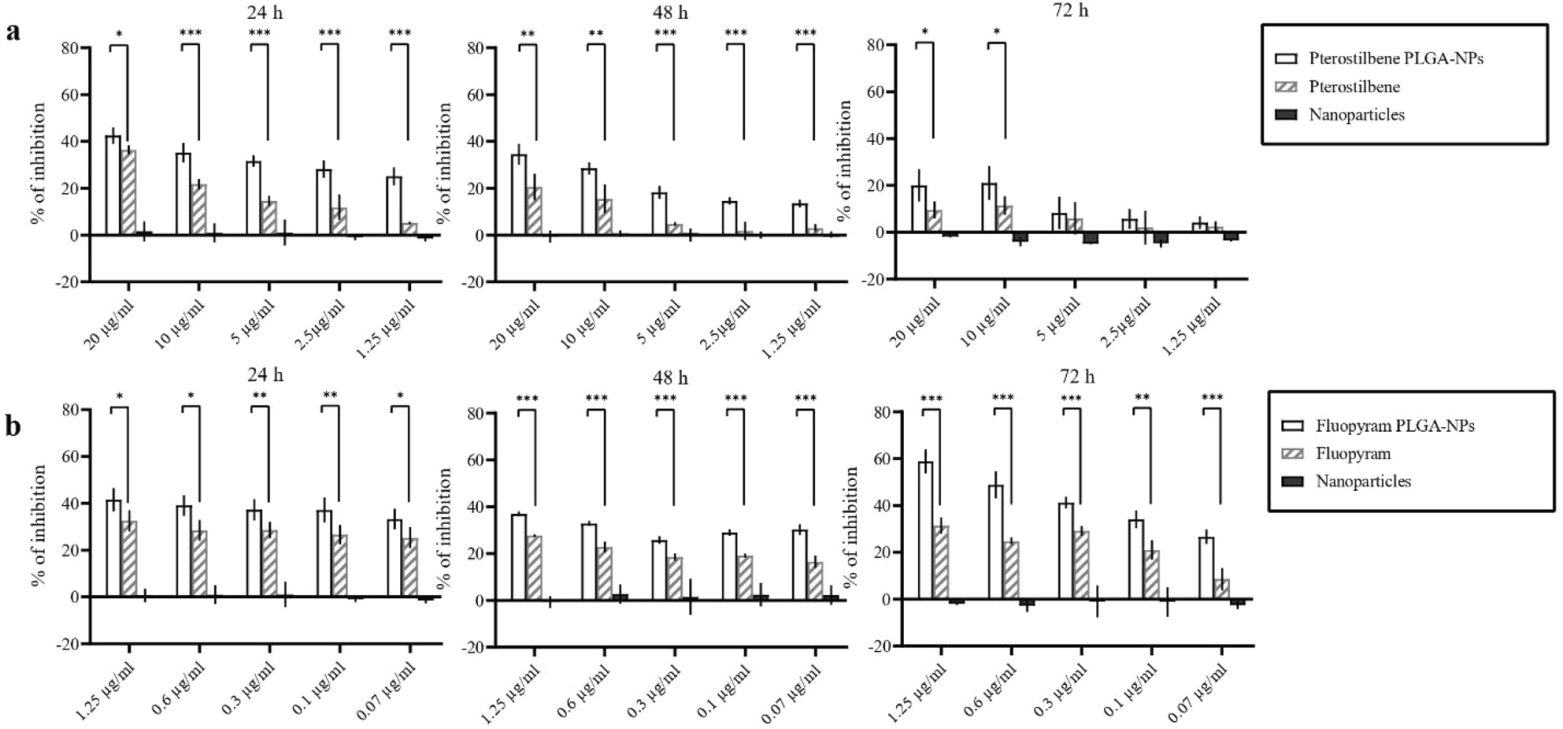
Activity of pterostilbene and fluopyram, free or loaded in PLGA NPs, against non-germinated conidia of *Botrytis cinerea* DSM 877 after 24, 48, and 72 h of incubation. *P ˂ 0.05, **P ˂ 0.01, ***P ˂ 0.001.

**Figure 5:**
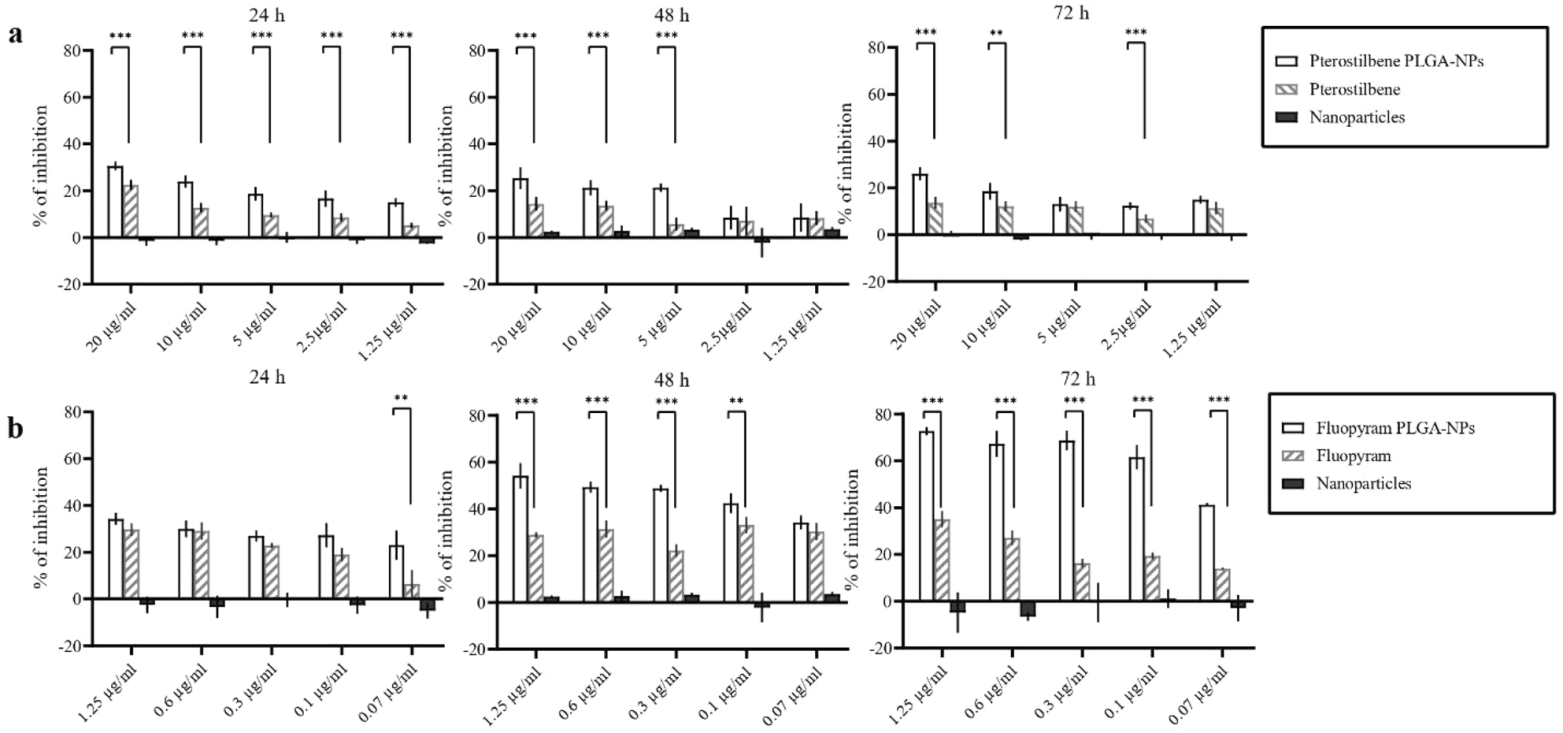
Activity of pterostilbene and fluopyram, free or loaded in PLGA NPs, against 12 h -germinated conidia of *Botrytis cinerea* DSM 877 after 24, 48, and 72 h of incubation. *P ˂ 0.05, **P ˂ 0.01, ***P ˂ 0.001.

In vitro antifungal activity of pterostilbene or fluopyram, free or loaded into PLGA NPs, against germinated and non-germinated conidia has been determined by evaluating the metabolic activity of fungal cells after 1, 5, 24, 48 and 72 h of incubation. No statistically significant differences were observed after 1 and 5 h of incubation between NPs loaded with pterostilbene or fluopyram and the free compounds (data not shown). Conversely, after 24 and 48 h of incubation with NPs loaded with pterostilbene or fluopyram, the metabolic activity of the conidia was lower compared to when these compounds were administered in their free form, as shown in Fig. 4. These data demonstrated that after 24 and 48 h of incubation both fluopyram or pterostilbene loaded into NPs showed better antifungal activity than the free compounds (Fig. 4). In both experiments, empty NPs did not show antifungal activity. NPs loaded with pterostilbene, added to germinated conidia, showed significant inhibition of *B. cinerea* growth after 24 h of incubation. NPs loaded with fluopyram significantly inhibited *B. cinerea* growth, compared to free fluopyram, after 72 h of incubation, in all tested concentrations (Fig. 5). The antifungal activity of pterostilbene, a well-known plant phytoalexin, on *B. cinerea* has already been demonstrated by Pezet and Pont since 1990^32^. More recently, Xu and collaborators^21^ reported 45% of mycelial growth reduction after treatment of *B. cinerea* with 200 µg mL^−1^ of pterostilbene. Moreover, Xu and collaborators^33^ in a subsequent study showed multiple action mechanisms of pterostilbene against *B. cinerea*. After treatment with pterostilbene, *B. cinerea* changed the morphology of the hyphae and conidiophores, lost plasma membrane integrity, modulated gene expression, and increased laccase production. Some researchers reported that *B. cinerea*, through laccase secretion, can degrade and neutralize the toxicity of pterostilbene^34^. Our results show a significant and long lasting increase in activity, even at low concentrations. The significant increase in activity of pterostilbene delivered by PLGA NPs could be due to the protection of pterostilbene from fungal laccase. Moreover, the higher hydrophobicity of pterostilbene delivered by PLGA NPs could increase diffusion through fungal membranes. On the other hand, pterostilbene with multiple mechanisms of action carries a low risk of developing resistance and its low toxicity makes pterostilbene a winning compound to fight *B. cinerea*. Synthetic fungicides are currently widely used in agriculture to fight *B. cinerea* infections. In this context, fluopyram, a synthetic pyridinyl ethylbenzaimide fungicide that inhibits succinate dehydrogenase (SDH), is commonly used to safeguard crops. Some studies reported high risks of *B. cinerea* resistance development to fluopyram and other succinate dehydrogenase inhibitors^35^. However, Dong and Hu^36^ have shown that the transformation products were more toxic than fluopyram. Moreover, fluopyram shows high residue in surface soil which affects subsequent crops^37^. In this study, NPs loaded with fluopyram significantly inhibited *B. cinerea* growth, compared to free fluopyram.

Agriculture is the backbone of the economy of most countries and it was the key development in the rise of human civilization. *B. cinerea* is a well-known fungus with a wide host range that causes heavy losses in crop yields every year. The generation of drug delivery systems based on nanomaterials represents a potential alternative to developing newer formulations to successfully control fungal infections and overcome the fungal multi-resistance to existent drugs. In fact, the encapsulation protects the antifungal compound from damage such as photolysis or degradation, allowing the drug to reach the target site more efficiently with a consequent reduction in the number of applications^38,39^. In this context, the delivery of antifungal compounds encapsulated in PLGA NPs could be considered an excellent strategy to safeguard crops against *B. cinerea.* The fluorescence microscopy observations revealed that 50 nm Cu6-PLGA NPs penetrated *B. cinerea* conidia and hyphae as early as 10 min after administration. Moreover, the antifungals pterostilbene and fluopyram delivered by NPs exhibited higher antifungal activity against *B. cinerea* than the corresponding antifungal compounds administered in free form. These results lay the foundation for the use of PLGA NPs in agriculture as a new strategy in plant pest management with the goal of enhancing the effectiveness of natural and synthetic antifungals through controlled and targeted drug delivery in the agronomic field.
